# Comparison of immunogenicity and vaccine efficacy between heat-shock proteins, HSP70 and GrpE, in the DnaK operon of *Mycobacterium tuberculosis*

**DOI:** 10.1038/s41598-018-32799-z

**Published:** 2018-09-26

**Authors:** Woo Sik Kim, Jong-Seok Kim, Hong Min Kim, Kee Woong Kwon, Seok-Yong Eum, Sung Jae Shin

**Affiliations:** 1 Department of Microbiology, Institute for Immunology and Immunological Disease, Brain Korea 21 PLUS Project for Medical Science, Yonsei University College of Medicine, Seoul, South Korea; 2 Advanced Radiation Technology Institute, Korea Atomic Energy Research Institute, Jeongeup, South Korea; 3 Division of Immunopathology and Cellular Immunology, International Tuberculosis Research Center, Changwon, South Korea; 4 Present Address: Myunggok Medical Research Institute, College of Medicine, Konyang University, Daejeon, South Korea

## Abstract

Antigens (Ags) in *Mycobacterium tuberculosis* (Mtb) that are constitutively expressed, overexpressed during growth, essential for survival, and highly conserved may be good vaccine targets if they induce the appropriate anti-Mtb Th1 immune response. In this context, stress response-related antigens of Mtb might serve as attractive targets for vaccine development as they are rapidly expressed and are up-regulated during Mtb infection *in vivo*. Our group recently demonstrated that GrpE, encoded by *rv0351* as a cofactor of heat-shock protein 70 (HSP70) in the DnaK operon, is a novel immune activator that interacts with DCs to generate Th1-biased memory T cells in an antigen-specific manner. In this study, GrpE was evaluated as a subunit vaccine in comparison with the well-known HSP70 against the hyper-virulent Mtb Beijing K-strain. Both HSP70- and GrpE-specific effector/memory T cells expanded to a similar extent as those stimulated with ESAT-6 in the lung and spleen of Mtb-infected mice, but GrpE only produced a similar level of IFN-γ to that produced by ESAT-6 stimulation during the late phase and the early phase of Mtb K infection, indicating that GrpE is highly-well recognised by the host immune system as a T cell antigen. Mice immunised with the GrpE subunit vaccine displayed enhanced antigen-specific IFN-γ and serum IgG2c responses along with antigen-specific effector/memory T cell expansion in the lungs. In addition, GrpE-immunisation markedly induced multifunctional Th1-type CD4^+^ T cells co-expressing IFN-γ, TNF-α, and IL-2 in the lungs of Mtb K-infected mice, whereas HSP70-immunisation induced mixed Th1/Th2 immune responses. GrpE-immunisation conferred a more significant protective effect than that of HSP70-immunisation in terms of bacterial reduction and improved inflammation, accompanied by the remarkable persistence of GrpE-specific multifunctional CD4^+^ T cells. These results suggest that GrpE is an excellent vaccine antigen component for the development of a multi-antigenic Mtb subunit vaccine by generating Th1-biased memory T cells with multifunctional capacity, and confers durable protection against the highly virulent Mtb K.

## Introduction

Although the prevention of tuberculosis (TB) is the most effective control measure to reduce the incidence of TB, the protection efficacy of bacillus Calmette-Guerin (BCG), the only currently available licensed TB vaccine^1^, is thought to be insufficient to protect against pulmonary TB and latent infection. In addition, variable results of BCG vaccine efficacy for different geographical locations have been reported because *Mycobacterium tuberculosis* (Mtb) genotypes with different virulence levels may be dominant in different regions^2–4^. Importantly, the Mtb Beijing genotype is highly prevalent in East Asian countries including China, Korea, and Japan and the isolation rate of strains belonging to the Mtb Beijing family has increased worldwide, indicating that BCG vaccine provides a relatively low level of protection against this Mtb genotype^5,6^. Furthermore, epidemiological studies have recently suggested that continuous BCG vaccination may have driven the emergence of the Beijing genotype^6^. Thus, the control of Mtb Beijing strains is a major challenge and is urgently needed globally.

To develop new prophylactic vaccines capable of replacing and/or boosting the BCG vaccine, many vaccine candidates have entered into the clinical^7,8^. In particular, prophylactic vaccines require Ag targets that are expressed during the early phase of infection and are recognised by the host immune system to immediately initiate host defense mechanisms^9,10^.

It is well documented that host defense against Mtb infection is strongly associated with the expansion and generation of Mtb-specific multifunctional Th1-type T cell subsets, in concert, to activate macrophages, thereby limiting mycobacterial replication during the early infection period^11,12^. Thus, the identification of Ags triggering protective T cell subsets at the early infection course is a goal of TB vaccine development. In this regard, Ags in Mtb that are constitutively expressed, overexpressed during growth, essential for survival and growth, and highly conserved may be good vaccine targets if they induce a prompt anti-Mtb Th1 immune response^13,14^. Following this rationale, many Mtb vaccine Ag targets, including Ag85 complex antigens, ESAT-6 (early secreted anti-genic target-6), and heat shock proteins (HSPs), have been evaluated as target vaccine antigens because they are abundantly expressed and induce a strong cell-mediated immune defense response by evoking T-cell proliferation and IFN-γ production in an antigen-specific dependent manner, especially during the early phase of Mtb infection^15,16^.

Accordingly, stress response-related antigens of Mtb are attractive targets for vaccine development, as they are rapidly expressed and up-regulated during Mtb infection^17,18^. HSPs are essential molecular chaperones for the maintenance of cellular functions in normal as well as stress conditions^19^. The regulation of the expression of HSPs plays an important role in the pathogenesis of Mtb^20^. Among mycobacterial HSPs, Mtb HSP70 is the most well-known Ag linking innate and adaptive immune responses with potent adjuvant activity^21^. In Mtb, the *hsp70* operon consists of the *dnaK*-*grpE*-*dnaJ* (*rv0350*-*rv0351*-*rv0352*) genes and is co-expressed and negatively regulated by the repressor *hspR* (*rv0353*)^22^. The Dnak operon in mycobacterial species is essentially required for cell growth, even in the absence of stress, indicating that it is a promising target for the development of antibiotics and vaccines against mycobacteria^23^. Notably, due to the host immune response to HSPs, the constitutive over-expression of Mtb HSP70 results in the reduced survival of Mtb during prolonged infection^24^, indicating that it may be possible to design interventions capable of promoting an immediate and durable host immune response to control Mtb.

Although the role of HSP70 in the immune response to Mtb infection has been studied extensively, relatively little is known about its cofactor, GrpE, especially with respect to its immunological functions and TB vaccine potential. In addition, HSP70 and GrpE were recently identified as potential vaccine candidates in an in silico analysis of open reading frames in the Mtb genome^25^. Importantly, our group recently showed that Th1-type T-cell immunity-biasing effect of GrpE by intercommunicating with dendritic cells^26^.

In the present study, the immunogenicity and the potential protective efficacy of stress antigens in HSP70 and its co-factor GrpE in the DnaK operon were assessed in a head-to-head format in a Mtb Beijing strain challenge model by delivery as subunit vaccines. We found that GrpE is rapidly recognised by the host immune system and produces a similar level of IFN-γ to that produced by ESAT-6-stimulation during Mtb K infection. Following the challenge with Mtb K, the GrpE-subunit vaccine showed an equivalent protective efficacy to BCG immunisation against Mtb K by rapidly recruiting and activating GrpE-specific multi-functional CD4^+^ T cells in the lungs pre- and post-challenge, along with the expansion of antigen-specific Th1 and effector/memory T cells in the lungs of Mtb-infected mice. Thus, our results suggest that GrpE is a possible component of future multi-antigenic vaccines and therefore is a candidate for a novel TB vaccine.

## Results

### Purification of recombinant proteins

**Figure 1:**
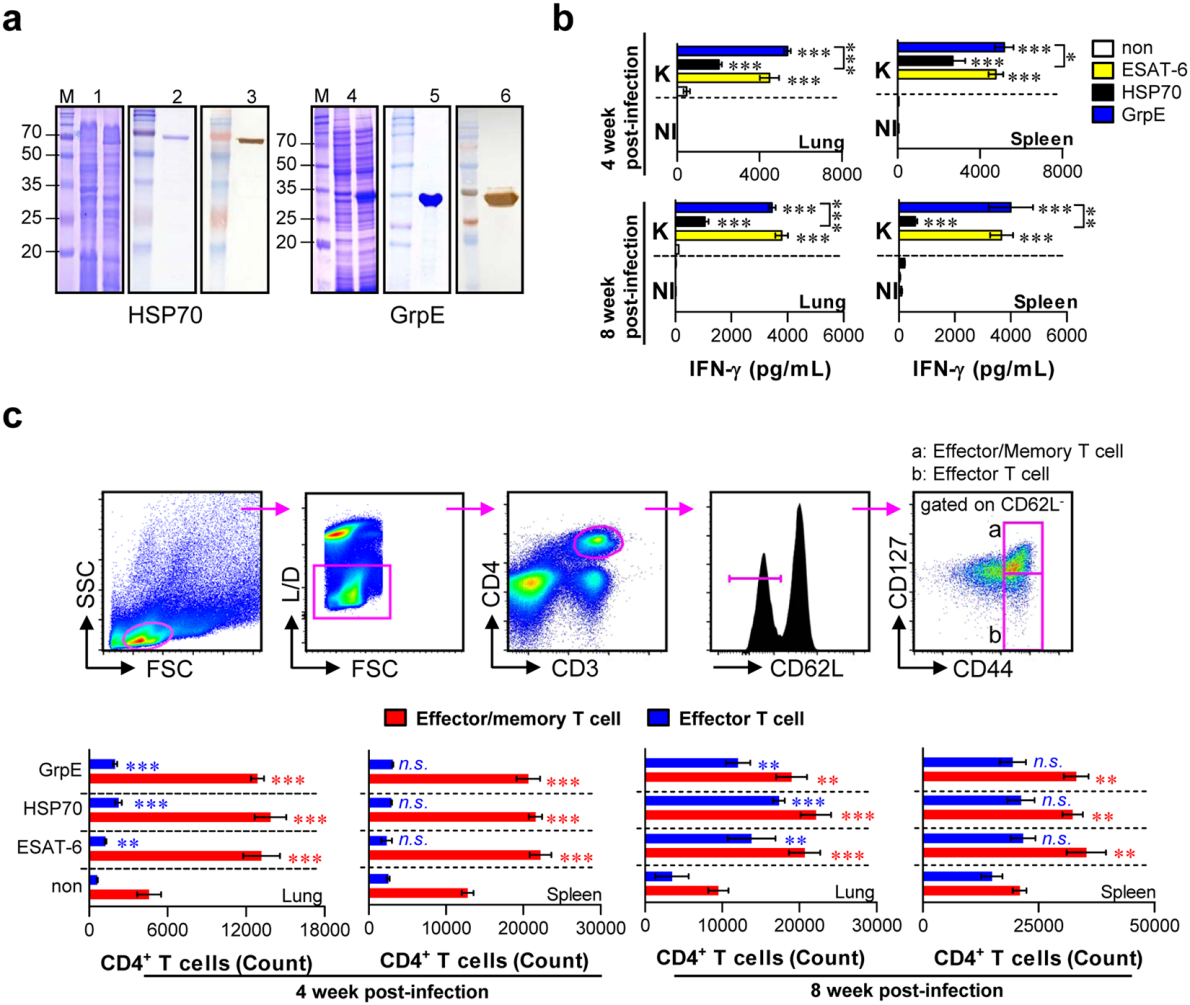
IFN-γ production and memory T cell phenotypes induced by recombinant antigen stimulation in the lung and spleen of Mtb K-infected mice. (**a**) HSP70 and GrpE antigens were successfully induced in *E*. *coli* BL21 by supplementation with 1 mM isopropyl β-d-thiogalactoside (IPTG) for 12 h. (**a**, 1 and 4) The expression of N-terminal His-tagged antigens was identified by SDS-PAGE (M, molecular weight markers; Lane 1, uninduced; Lane 2, induced). (**a**, 2 and 5) SDS–PAGE analysis of purified antigens by Ni-nitrilotriacetic acid (NTA). (**a**, 3 and 6) Western blot analysis of purified antigens using mouse anti-His antibodies. (**b**) HSP70 (10 μg/mL)- and GrpE (10 μg/mL)-specific IFN-γ production were measured in the spleen and lung cells of individual mice 4 and 8 weeks after challenge with aerosolised Mtb K strain (*n* = 4 mice/group). NI: Non-infected mice, K: Mtb K-infected mice. ESAT-6 (1 μg/mL) was used as a positive control. Data from one of two independent experiments are shown. (**c**) At the same time point, spleen and lung cells from Mtb K-infected mice (*n* = 4 mice) were stimulated *in vitro* with antigens, and numbers of CD4^+^ effector/memory and effector T cells were analysed by flow cytometry. Data from one of two independent experiments are shown. All results are expressed as means ± SD and statistical significance (*p < 0.05, **p < 0.01, or ***p < 0.001) is shown for treatments compared to the non-treated each T cells (non; effector or effector/memory T cells, respectively). The value of *n*.*s*. was defined as no significant effect.

For the purification of recombinant proteins, HSP70 and GrpE were expressed with a 6× histidine-tag in *E*. *coli* BL21 (Fig. 1a). Both recombinant proteins were purified as soluble forms; SDS-PAGE revealed that the molecular weights of HSP70 and GrpE were around 70 kDa and 32 kDa, respectively. The purified protein showed the expected molecular mass based on SDS-PAGE. Protein purification was ultimately confirmed by Western blotting. The endotoxin contents were measured by an LAL assay and were undetectable by showing below 10 pg/mL (<0.1 EU/mL) in both antigen preparations. These results indicate that HSP70 and GrpE were successfully purified for subsequent experiments.

### HSP70 and GrpE induced an antigen-specific IFN-γ response and effector/memory T cell expansion in Mtb K-infected mice upon *ex vivo* re-stimulation

Many intracellular bacterial infections induce memory T cell and antigen-specific T cell responses by bacterial antigens^27^. Prior to evaluating the efficacy of the two vaccine antigen candidates, we investigated whether HSP70 and GrpE recognition by the host immune system can induce an immunological T cell activation along with memory response during the course of Mtb infection^9^. We therefore analysed the antigen-specific IFN-γ response (Fig. 1b) and the generation of effector or effector/memory CD4 T cells (Fig. 1c) by HSP70, GrpE, or ESAT-6 (as a positive control) stimulation in T cells of Mtb-infected mice. Single cell suspensions from the lung and spleen of Mtb K*-*infected mice were prepared at 4 and 8 weeks post-infection. The cells were stimulated with HSP70 and GrpE for 24 h, and IFN-γ production was analysed by ELISA (Fig. 1b). As shown in Fig. 1b, lung and spleen cells from the Mtb K*-*infected mice show higher levels of IFN-γ in response to GrpE stimulation compared to those for HSP70 stimulation. Also, we measured effector (CD44^+^CD127^+^CD62L^−^ cells) and effector/memory (CD44^+^CD127^+^CD62L^+^ cells) via the surface expression of CD44 and CD127 on CD3^+^CD4^+^-gated CD62L^−^ by flow cytometry (Fig. 1c; top panel). Both HSP70 and GrpE stimulation substantially increased effector/memory T cells, but there was no difference between HSP70 and GrpE stimulation (Fig. 1c; bottom panel). Taken together, HSP70 and GrpE are immunologically recognised during Mtb K infection; thus, Ag-specific memory T cells are generated.

### Immunogenicity and immunological memory induced by HSP70 and GrpE immunisation

**Figure 2:**
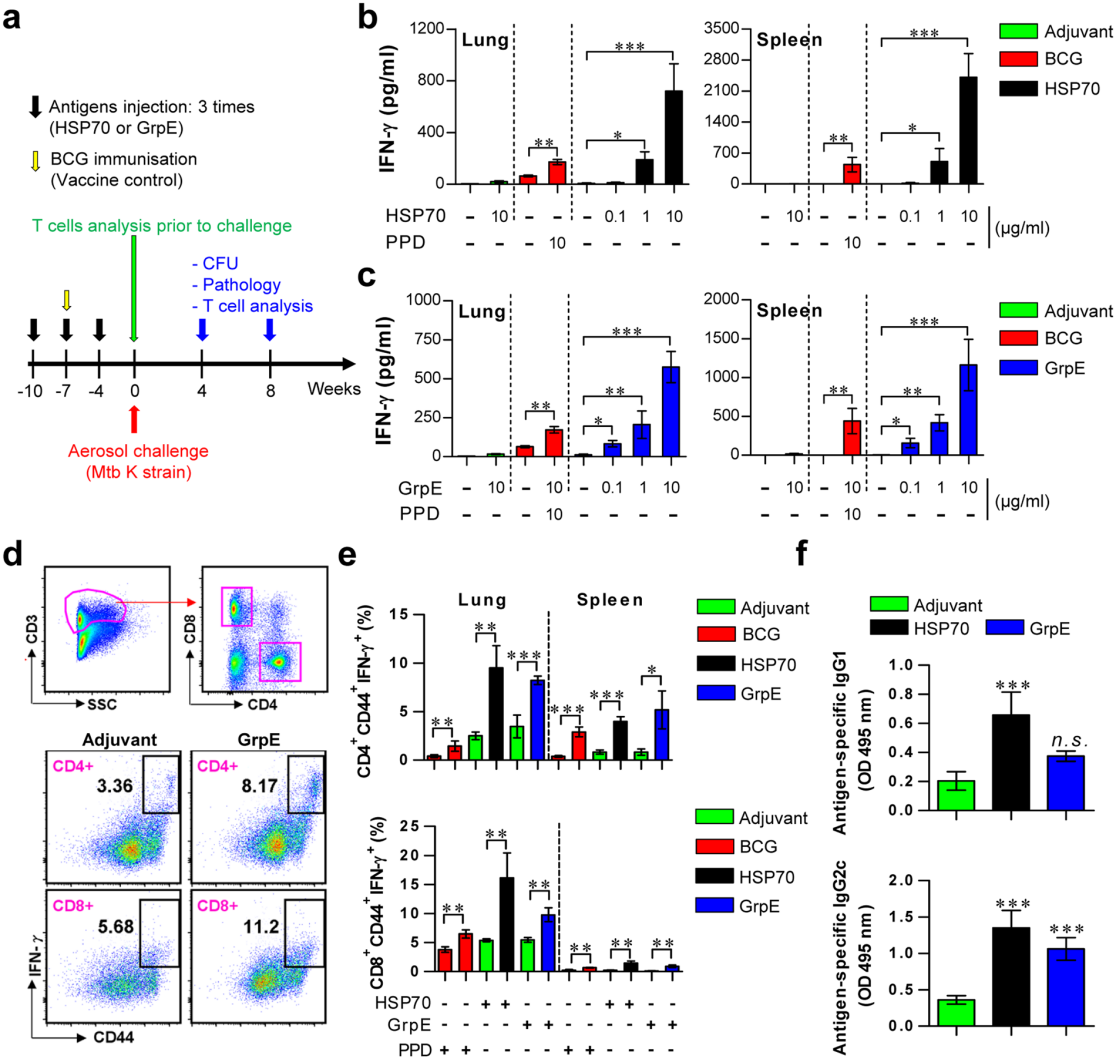
Immunogenicity in the lung and spleen of HSP70- and GrpE-immunised mice. (**a**) Schematic diagram of the subunit vaccine experimental design. (**b**,**c**) Mice (*n* = 5 mice/group) were immunised with BCG (Red bars), HSP70 (Black bars), GrpE (Blue bars), or adjuvant alone (Green bars), and their spleen and lung cells were stimulated *in vitro* with no antigen, PPD, HSP70, or GrpE 4 weeks after the last vaccination. IFN-γ secretion by spleen and lung cells in response to PPD (**b**,**c**), HSP70 (**b**) and GrpE (**c**) stimulation was analysed by ELISA. (**d**) An example of the gating strategy for the analysis of antigen-specific IFN-γ producing T cells among lung T cells of one representative mouse from the GrpE-immunised group is shown. (**e**) The percentages of PPD-, HSP70- or GrpE-specific IFN-γ-producing CD3^+^CD4^+^CD44^high^ and CD3^+^CD8^+^CD44^high^ T cells were analysed in cells isolated from the spleen and lungs of BCG- and antigen-immunised mice (*n* = 5 mice/group). (**f**) Serum levels of antigen-specific IgG1 and IgG2c at 4 weeks after the final immunisation (*n* = 5 mice/group). Data from one of two independent experiments are shown. *p < 0.05, **p < 0.01, or ***p < 0.001, compared to the adjuvant control group (Green bars).

Incomplete Freund’s adjuvant (IFA), which lacks mycobacterial components, was used in this study. We first investigated the IFN-γ response of T cells after BCG and IFA-based antigen immunisation. After the final immunisation, we assessed IFN-γ responses (Fig. 2b,c), as well as the up-regulation of antigen-specific IFN-γ-producing memory Th1 cells (Fig. 2d,e) upon cognate antigen stimulation in the spleen and lung cells of immunised mice. Purified protein derivative (PPD) was used as a stimulant for the T cells of the BCG vaccine-immunised mice. Antigen-specific IFN-γ^+^ T cells were evaluated by T cell specific markers (CD3^+^CD4^+^ and CD3^+^CD8^+^) and activated or a memory Th1 cell marker (CD44^high^) using flow cytometry (Fig. 2d). BCG-, HSP70- and GrpE-immunisation induced IFN-γ release (Fig. 2b,c) and the number of IFN-γ^+^CD44^high^CD4^+^ and CD8^+^ T cells (Fig. 2e) in response to re-stimulation with PPD, HSP70, and GrpE were measured. A further analysis of the antigen-specific IgG1 response, an indicator of the Th2 response, showed that the HSP70-immunised groups exhibited a HSP70-specific IgG1 response. Additionally, all groups exhibited an elevated IgG2c response, an indicator of the Th1 response, compared with that of adjuvant control groups (Fig. 2f).

### Protective efficacy of the antigen vaccine in the Mtb K-infected mouse model

**Figure 3:**
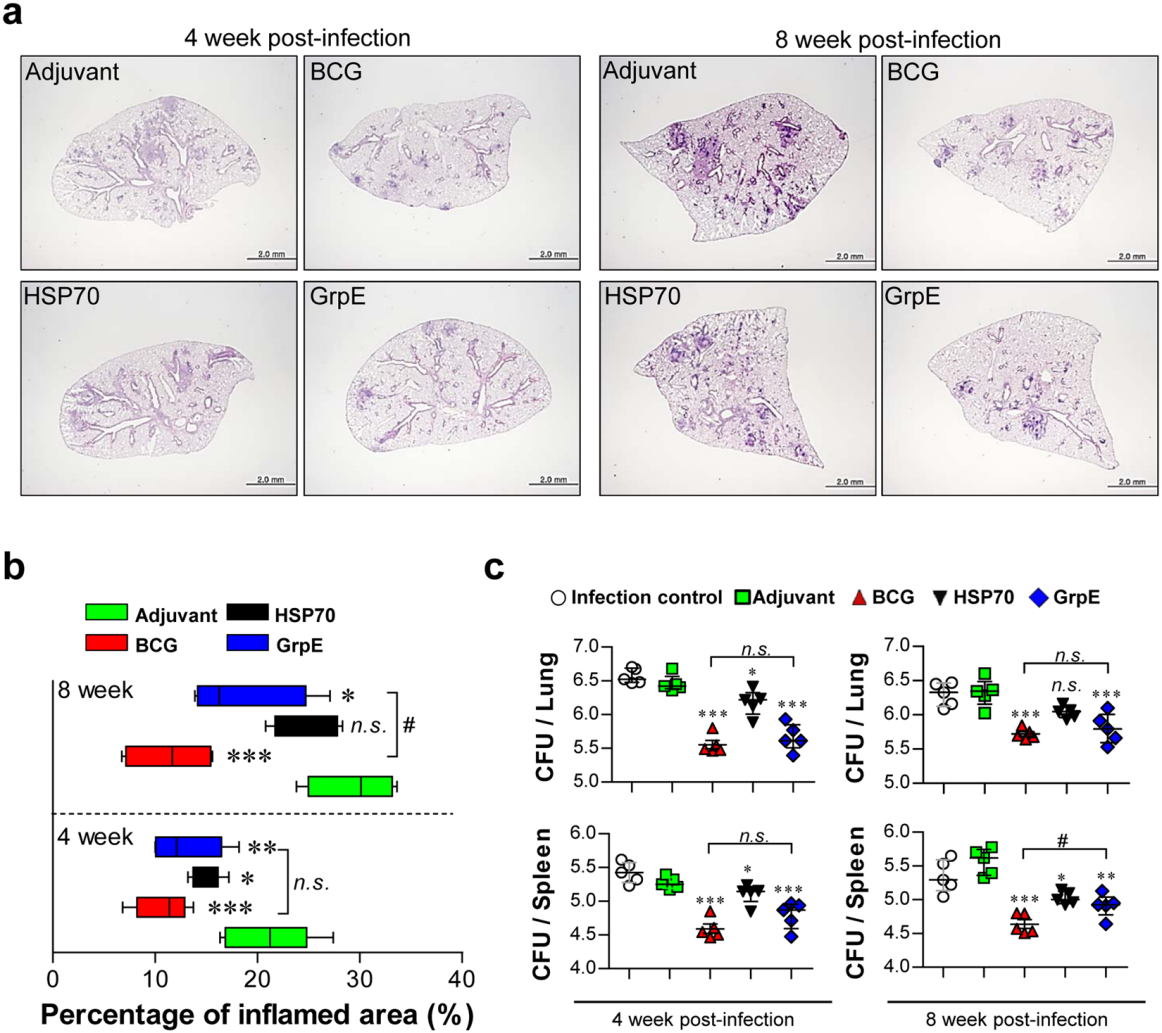
Protective efficacy of antigen immunisation against Mtb K infection. (**a**) H&E-stained sections (*n* = 5 mice/group) of the lung for each immunised mouse (Adjuvant alone, BCG, HSP70, or GrpE) at 4 and 8 weeks after challenge with the aerosolised Mtb K strain. One BCG vaccinated group was included as a positive control. (**b**) Lung inflammation values are presented as the mean percentages of the area of inflammation from lung sections of infected mouse groups. (**c**) The differences in bacterial numbers between vaccinated groups and adjuvant control groups at 4 and 8 weeks after challenge are shown. Data from one of two independent experiments are shown. *p < 0.05, **p < 0.01, or ***p < 0.001, when compared to adjuvant control group; green bars or plots (a one-way ANOVA followed by Dunnett’s test). ^#^*p* < 0.05 (BCG *vs*. GrpE; unpaired *t*-test).

Four weeks after the last immunisation, the ability of protein vaccines to protect against Mtb K was analysed by aerosol challenges. For the histopathological analysis, lungs were isolated and stained with H&E at 4 and 8 weeks post-infection. The lungs of adjuvant alone-, HSP70-, GrpE-, and BCG vaccine-immunised groups (as a positive control for the efficacy testing of TB vaccines in this study) were analysed with respect to histology (Fig. 3a,b) and bacterial burden (Fig. 3c). Lung tissues from GrpE- and BCG vaccine-immunised mice 4 and 8 weeks after Mtb K challenge clearly showed reduced granulomatous inflammation compared to the lung tissues of the adjuvant control group, along with the microscopic appearance (Fig. 3b). However, a reduction of granulomatous inflammation via HSP70 immunisation was only observed at 4 weeks post-infection. In addition, with respect to bacterial burden, the group immunised with GrpE showed a significant reduction of CFUs in the lung and spleen at 4 and 8 weeks after Mtb K challenge compared to the adjuvant alone group. HSP70 immunisation had a minor effect on the bacterial burden in lung and spleen (Fig. 3c). However, 8 weeks post the Mtb K challenge, the degree of pathological improvement observed after GrpE vaccination was lesser than that observed after BCG immunisation (Fig. 3b).

### GrpE immunisation dominantly increases the Th1 immune response during the course of Mtb K infection, while HSP70 immunisation simultaneously induces mixed Th1/Th2 responses

**Figure 4:**
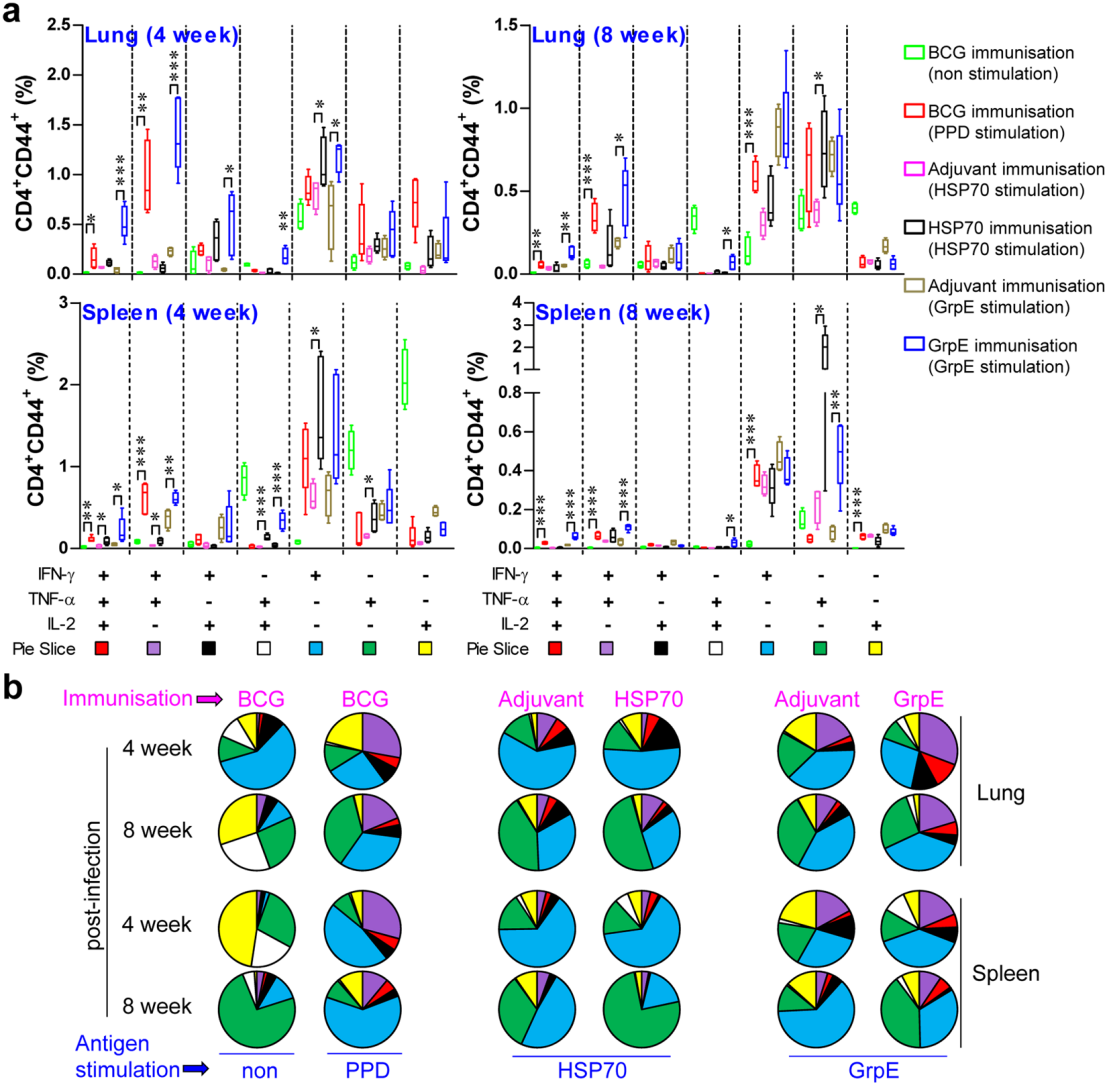
Antigen-specific multifunctional CD4^+^ T cell responses in antigen-immunised mice after challenge with Mtb K. BCG-, HSP70-, GrpE-, or adjuvant alone-immunised mice were infected by aerosol exposure to Mtb K. At different time points after infection, as indicated, mice (*n* = 5 mice/group at 4 and 8 weeks post-infection) were euthanised and their lung cells (**a** and **b**; top panel) and splenocytes (**b**; bottom panel) were stimulated *in vitro* with PPD (10 μg/mL), HSP70 (10 μg/mL) or GrpE (10 μg/mL). (**a**) The percentage of antigen-specific CD4^+^CD44^high^ T cells producing IFN-γ, TNF-α, or IL-2 was measured by use of the gating strategy shown in Supplementary Fig. S1, in cells isolated from lung cells and splenocytes at 4 and 8 weeks after challenge with Mtb K strain. (**b**) The pie charts present the mean frequencies of cells co-expressing IFN-γ, TNF-α, and/or IL-2. The cytokine profiles for individual cells were analysed by multi-colour flow cytometry by gating for lymphocytes, CD3^+^CD4^+^CD44^high^. Data from one of two independent experiments are shown. *p < 0.05, **p < 0.01, or ***p < 0.001 relative to the control groups, respectively.

**Figure 5:**
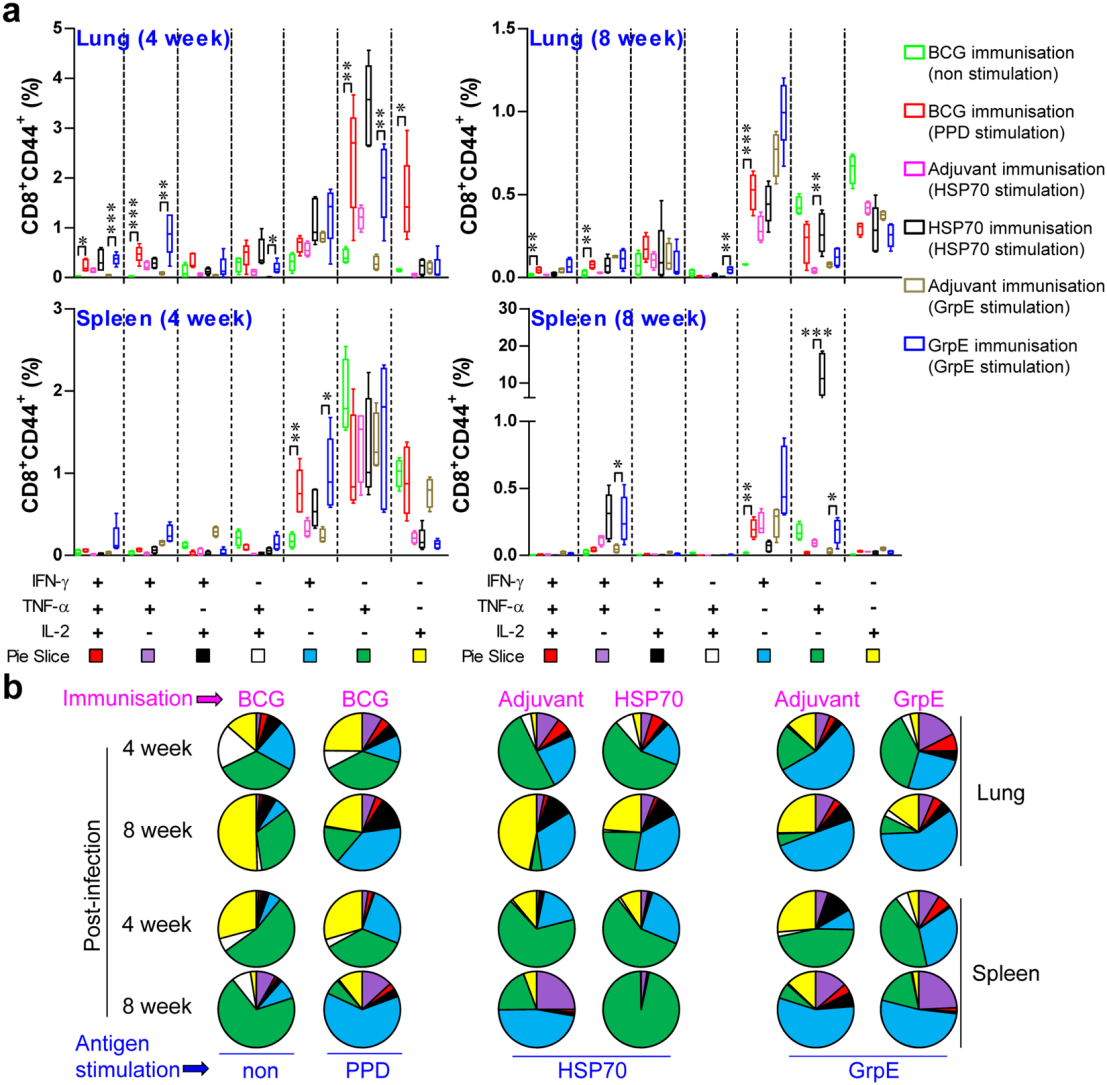
Antigen-specific multifunctional CD8^+^ T cell responses in antigen-immunised mice after Mtb K challenge. (**a**) The percentage of antigen-specific CD8^+^CD44^high^ T cells producing IFN-γ, TNF-α, or IL-2 measured in cells isolated from the lung (**a** and **b**; top panel) and splenocytes (**a** and **b**; bottom panel) of vaccinated mice (*n* = 5 mice/group at 4 and 8 weeks post-infection) at 4 and 8 weeks after challenge with Mtb K. The pie charts present the mean frequencies of cells co-expressing IFN-γ, TNF-α, and/or IL-2. The cytokine profiles for individual cells were analysed by multi-colour flow cytometry by gating for lymphocytes, CD3^+^CD8^+^CD44^high^. Data from one of two independent experiments are shown. *p < 0.05, **p < 0.01, or ***p < 0.001 relative to the control groups, respectively.

**Figure 6:**
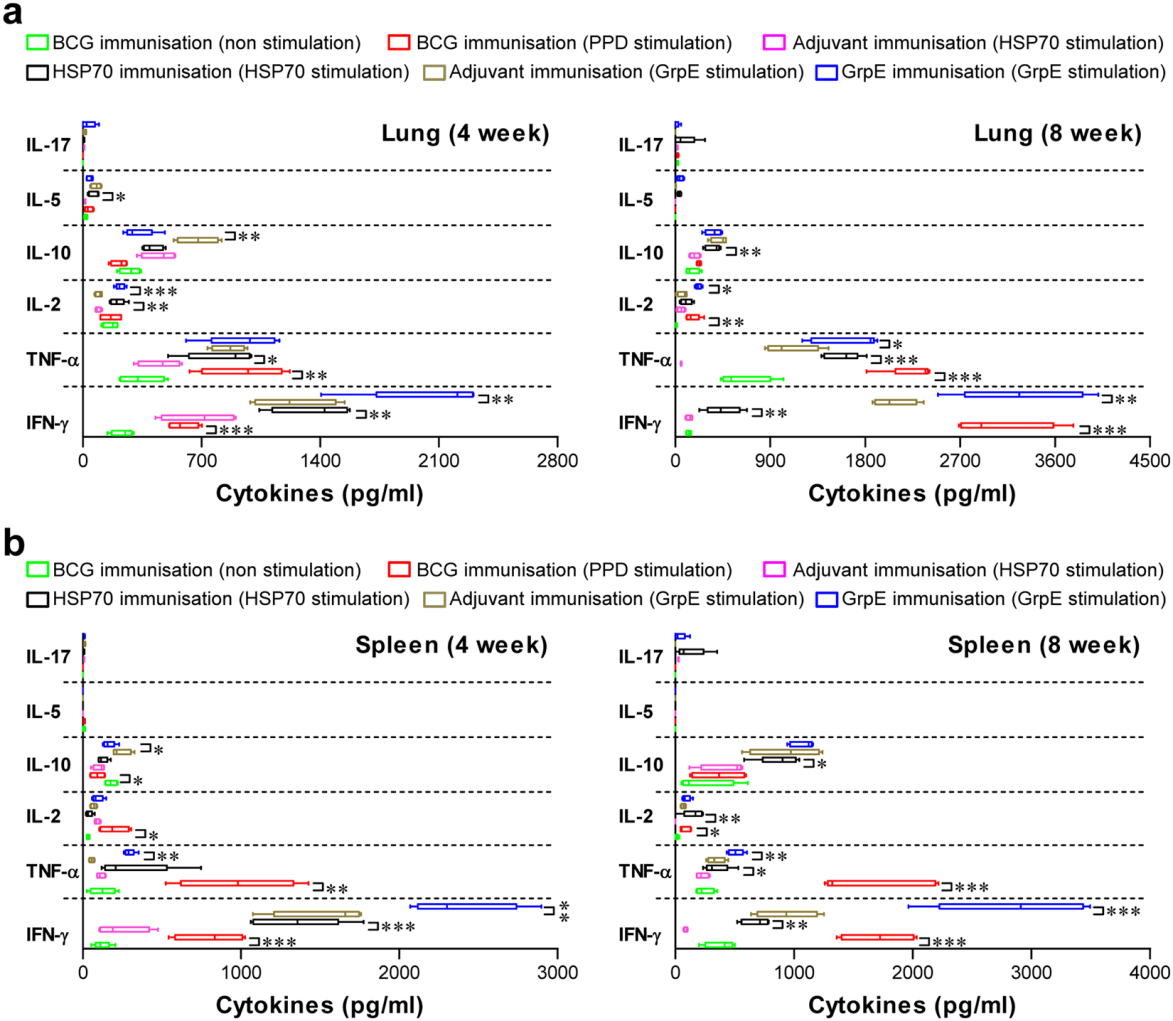
T cell immunity induced by antigen immunization. (**a**,**b**) Spleen and lung cells from BCG-, adjuvant alone-, HSP70-, and GrpE-immunised mice (*n* = 5 mice/group at 4 and 8 weeks post-infection in each group) were stimulated with PPD, HSP70, or GrpE, and the supernatant was collected and assayed for cytokines by ELISA. Antigen-specific cytokine responses are shown for spleen and lung cells collected post-infection (4 and 8 weeks). Data from one of two independent experiments are shown. *p < 0.05, **p < 0.01, or ***p < 0.001 by unpaired *t*-tests.

Because a decrease in multifunctional T cells is associated with increased bacterial growth in active Mtb*-*infection models^28^, both the Th1-type immune response and multifunctional T cells may be potential correlates of an effective host-protective immune response in TB^29^. Base on established the methodology from the previous study for multiufnctionality of T cells^30^, we next assessed the T cell phenotype and immune responses that are induced following antigen vaccination. At 4 and 8 weeks after the challenge, lung cells (top panel of Figs 4a and 5a) and splenocytes (bottom panel of Figs 4a and 5a) were stimulated *in vitro* with PPD, HSP70 or GrpE and the phenotypes of CD4^+^ (Fig. 4) and CD8^+^ (Fig. 5) antigen-specific T cells were evaluated by multi-colour intracellular cytokine staining and flow cytometry (Supplementary Fig. S1). Culture supernatants containing the secreted cytokines were collected from splenocytes and lung cells after antigen re-stimulation and analysed by ELISA (Fig. 6). As a consequence, the groups immunised with BCG, HSP70, or GrpE exhibited robust responses against their respective antigens after re-stimulation. Interestingly, increased levels of triple-positive (IFN-γ^+^TNF-α^+^IL-2^+^) and double-positive (IFN-γ^+^TNF-α^+^) antigen-specific CD4^+^CD44^high^ T cells were observed in the BCG-, GrpE-immunised group compared to the control groups in the spleen and lung at 4 and 8 weeks post-infection (Fig. 4), and triple-positive (IFN-γ^+^TNF-α^+^IL-2^+^) and double-positive (IFN-γ^+^TNF-α^+^ and TNF-α^+^IL-2^+^) antigen-specific CD8^+^CD44^high^ T cells were observed in the lung at 4 weeks post-infection. In the BCG-immunised groups, the cells were well-maintained for up to 8 weeks post infection (Fig. 5). However, in the case of the HSP70-immunised group, cytokine-secreting CD4^+^ and CD8^+^ T cells were significantly decreased in the lung and spleen compared with those of the BCG- or GrpE-immunised group (Figs 4 and 5). Furthermore, the lung and spleen of mice immunised with HSP70 induced Th1-related cytokines together with Th2-related cytokines, such as IL-5 and IL-10 (Fig. 6a), whereas BCG and GrpE-immunisation induced a significantly higher frequency of Th1-related cytokines, including TNF-α, IFN-γ, and IL-2 (Fig. 6b). Interestingly, antigen-specific IgG1 and IgG2c responses did not differ significantly between adjuvant control groups and antigen-immunised groups after Mtb infection (data not shown). These findings suggest that GrpE immunisation induces a greater Th1 response than HSP70 immunisation, but not a humoral response.

## Discussion

Inside host cells such as macrophages and dendritic cells, Mtb may experience many harsh conditions. In particular, Mtb Ags play important roles in cell-to-cell communication, detoxification, eliciting host immune responses, and essentially aiding in Mtb growth. They are of great interest as vaccine antigen targets because their expression is obligatory for Mtb survival and they are likely to be recognised by host immune cells.

Overall, effective Ag targets for the rational design of Mtb vaccines should satisfy the following requirements: constitutive expression, accessibility (cell wall-associated or secreted) to Ag-presenting cells (APCs), recognised by the immune system during the early infection phase *in vivo*, ability to induce a Th1-biased memory immune response, and eventual efficacy against hyper-virulent Mtb strains. In this study, we evaluated antigens in the DnaK operon as vaccine candidates that fulfil these criteria and are effective against challenge with the highly virulent Mtb K strain in a head-to-head comparison format.

In vaccine design, early protection by the rapid recruitment of IFN-γ-producing CD4^+^ effector T cells to the infected site is crucial because once acquired immunity is initiated, Mtb growth is inhibited^31^. For example, ESAT-6 is recognised promptly by T cells with strong immunogenicity at the early phase of infection and is a major antigen component of many prophylactic vaccine candidates^16,32^. However, it is believed that ESAT-6 is a key factor in diagnostic tools^33,34^, indicating that ESAT-6-based vaccines may be difficult for differentiation between naturally infected subjects and vaccinated people. Thus, a novel T cell antigen recognised by the host immune system at the early phase of infection is required for TB vaccine development, without interfering with diagnosis. In the present study, we first investigated whether HSP70 and GrpE are recognised by the host immune system during the early (4 weeks) and late (8 weeks) phases of Mtb infection by comparisons with ESAT-6. In our study, IFN-γ production (Fig. 1b) and CD4^+^ memory T cell expansion (Fig. 1c) were observed in spleen and lung cells isolated from mice treated with HSP70 and GrpE at various time points after infection with Mtb K, indicating that both antigens are recognised by the host immune system as immunogenic antigens. In particular, GrpE exhibited a comparable IFN-γ response to that of ESAT-6, the most well-characterised immunogenic Mtb Ag at the active phase of Mtb infection^35^, suggesting that GrpE is promptly recognised by the host immune system.

In fact, *grpE* is an essential gene, as determined by Himar1-based transposon mutagenesis, in the H37Rv strain and is highly conserved in all Mtb strains, with only a single gene copy^36–38^. In addition to the constitutive overexpression of *grpE*, basal *grpE* expression is essentially required for the survival and growth of Mtb, indicating that the obligatory expression of GrpE occurs during Mtb infection. Moreover, the over-expression of GrpE was identified in the most prevalent clinical Mtb strains from South India under hypoxic culture conditions compared to aerated cultures, while other HSPs, including HSPX, GroEL2, and GroES, are more highly expressed than the expression in the laboratory-adapted strain H37Rv, indicating that GrpE is up-regulated during clinical Mtb infection and may have vaccine potential^39^.

In general, high CD44 expression on T cell surfaces indicates that the T cells are antigen-experienced (*i*.*e*., they exhibit an antigen-specific memory phenotype)^30,40^. In addition, a recent study has shown that CD44 is essential for the generation and maintenance of memory T helper 1 (Th1) cells^41^. Interestingly, HSP70- and GrpE-immunised mice showed increased populations of IFN-γ^+^CD44^high^ T cells in both the lung and spleen (Fig. 2e). Notably, both serum IgG1 and IgG2c, which are representative antibody isotypes for Th2 and Th1 responses^42^, respectively, increased in HSP70-immunised mice, but only the IgG2c antibody response increased significantly in GrpE-immunised mice (Fig. 2f). The C-terminal portion of the heat shock protein Hsp70 has an adjuvant function by stimulating Th1-polarizing cytokines in human monocytes to produce IL-12, TNF-α, NO, and C-C chemokines^43^. Furthermore, Mtb HSP70 induces the expression of IL-10 and inhibits T-cell proliferation *in vitro*, indicating that HSP70 has immunomodulatory properties, rather than inflammatory potential^44^.

Based on these results, we evaluated and compared the protective effects of HSP70 and GrpE subunit vaccines against the Mtb K strain in a mouse model. Interestingly, GrpE immunisation displayed a protective effect comparable to that of the BCG vaccine in terms of CFU reduction, while HSP70 showed marginal protection, indicating that GrpE showed better protection than HSP70 after challenge with the Mtb K strain based on assessments of lung pathology (Fig. 3a,b) and the reduction of bacterial CFUs (Fig. 3c). However, the mice in the BCG vaccination group showed a more substantial CFU reduction (spleen, 8 weeks post infection, *p* < 0.05) and attenuated lung inflammation, compared to the single GrpE-immunised mice (Fig. 3; lungs, 8 weeks post infection, *p* < 0.05). These results suggest that the live BCG vaccine contains many Ags that may augment the functions of a variety of agents of the protective T cell repertoire, including the antigen 85 complex^45,46^.

Recently, Forbes *et al*. showed that multifunctional abilities of T cells are associated with protection against Mtb infection in a mouse model^47^. In addition, a continuous decrease in the multifunctionality of T cells corresponds with a decrease in protection against challenge with Mtb in the mouse model^29^. Thus, we used multi-parameter flow cytometry to analyse CD4^+^ and CD8^+^ T cells at infectious site after immunisation. In this study, the GrpE immunisation remarkably induced a high frequency of multifunctional CD4^+^CD44^high^ T cells (IFN-γ^+^TNF-α^+^IL-2^+^, IFN-γ^+^TNF-α^+^, and TNF-α^+^IL-2^+^) in the spleen and lung, while less induction was observed in the HSP70-immunised group (Figs 4 and 5). In addition, in GrpE-immunised mice, significantly higher levels of Th1-related cytokines were induced, including TNF-α, IFN-γ, and IL-2, while HSP70-immunised mice exhibited the induction of Th1-related cytokines along with Th2-related cytokines, such as IL-5 and IL-10 (Fig. 6). In general, the Th2 type immune response is reported to be harmful for immunity against Mtb infection. Aron *et al*. reported that the adoptive transfer of Th2 cells leads to an increase in CFUs of Mtb in the lung^48^. In addition, mixed Th1/Th2 immune responses occur in patients with active tuberculosis^49^. Thus, the mixed Th1/Th2 immune response and IgG1/IgG2c antibody responses and immune responses may be responsible for a relatively lower level of protection in the HSP70-immunised group; the robust Th1 immune response induced by GrpE immunisation was associated with a high level of protection against Mtb K infection.

Taken together, GrpE is recognised by the immune system at the early phase of infection and has the ability to induce a robust and durable Ag-specific multifunctional Th1-type T-cell memory response, conferring protective immunity and imparting significant protection in a murine model against the Mtb K strain. These results indicate that GrpE is a potential Ag target for the development of future multi-antigenic vaccines against Mtb, especially against highly virulent Mtb Beijing strains. Furthermore, the efficacy of a BCG priming vaccination combined with a GrpE subunit vaccine should be investigated to determine whether the GrpE subunit vaccine can boost the efficacy of the BCG vaccine.

## Methods

### Ethics statement and animals

All animal experiments were conducted following the guidelines provided by the Korean Food and Drug Administration (KFDA). The experimental procedures were evaluated and approved by the Institutional Animal Care and Use Committee (Permit Number: 2015-0041) of Yonsei University Health System (Seoul, Korea). After obtaining permission for the experiments, specific pathogen-free (SPF) female C57BL/6J mice at 6–7 weeks of age were purchased from Japan SLC, Inc. (Shizuoka, Japan) and maintained under barrier conditions in a ABSL-3 facility at the Avison Biomedical Research Center in Yonsei College of Medicine. The animals were fed a sterile commercial mouse diet and provided water ad libitum.

### Expression and purification of recombinant proteins

Cloning was conducted in *Escherichia coli* (*E*. *coli*) DH5α. *rv0350* and *rv0351* were amplified from Mtb H37Rv (ATCC 27294) genomic DNA by polymerase chain reaction (PCR). The *rv0350* primers were as follows: 5′-GGGCCCCATATGGCTCGTGCGGTCGGGATC-3′ (forward primer; the *Nde*I site is underlined) and 5′-GGGCCCAAGCTTCTTGGCCTCCCGGCCGTCGTC-3′ (reverse primer; the *Hind*III site is underlined). The *rv0351* primers were as follows: 5′-CGCCATATGGTGACGGACGGAAATCAAAAGC-3′ (forward primer; the *Nde*I site is underlined) and 5′-CCCAAGCTTACTGCCCGACGGTTCTGATTC-3′ (reverse primer; the *Hind*III site is underlined). The amplified PCR products were inserted into the pGEMT Easy Vector and sequences of the plasmids with *rv0350* and *rv0351* insertions were analysed. Finally, these genes were ligated into pET22b (Novagen, Madison, WI, USA) and transferred to *E*. *coli* BL21 cells.

### Expression and purification of proteins

The expression and purification of recombinant proteins were performed as described previously^50^. For protein expression, transformed *E*. *coli* (pET22b-*rv0350* and pET22b-*rv0351*) and vector controls (pET22b in *E*. *coli* BL21 cells) were incubated in LB broth supplemented with 50 μg/mL ampicillin and 1 mM isopropyl-β-d-thio-galactoside (IPTG) at 37 °C for 12 h. To confirm the protein expression and purification, each step was evaluated by 12% SDS-PAGE with Coomassie brilliant blue staining and immunoblotting using an anti-His antibody (Lab Vision, Fremont, CA, USA). To remove endotoxin contamination, the dialyzed proteins were incubated with polymyxin B-agarose (Sigma, St. Louis, MO, USA) and analysed using the limulus amebocyte lysate (LAL) test (Lonza, Lockland, ME, USA), according to the manufacturer’s instructions. The purity of antigens was quantified using Quantity One (Bio-Rad, Hercules, CA, USA); values were obtained by dividing the intensity per square millimeter of the HSP70- and GrpE-specific bands by that of all protein bands in the preparation lane.

### Bacteria and culture condition

Mtb H37Rv (ATCC 27294) was purchased from the American Type Culture Collection (ATCC, Manassas, VA, USA), and the Mtb K strain was obtained from the strain collections at the Korean Institute of Tuberculosis (KIT, Osong, Chungchungbuk-do, Korea). BCG (Pasteur 1173P2) was kindly provided by the Pasteur Institute (Paris, France). The mycobacterial strains used in this study were cultured as described previously^51^. Enumerated mycobacteria were used for subsequent experiments.

### Analysis of memory responses induced by antigen stimulation in T cells during the course of Mtb infection

C57BL/6 mice were aerogenically infected with Mtb K as previously described^52^. The bacteria were counted 1 day after aerosol exposure; approximately 150 viable bacteria were found to be delivered to the lungs. 4 and 8 weeks post infection, single-cell suspensions from the spleen and lungs of Mtb K-infected mice were prepared as described previously^14^. The single-cell suspensions were stimulated with antigens (HSP70, GrpE, and ESAT-6) for 24 h at 37 °C, and then, the cells and culture supernatants were examined for IFN-γ production and effector/memory T cell responses using ELISA assay and flow cytometric analysis, respectively.

### Immunisation and evaluation of vaccine efficacy in a murine model

For the adjuvant control and antigen vaccination groups, C57BL/6 J mice were immunised subcutaneously 3 times at 3-week intervals with incomplete Freund’s adjuvant (Sigma) (Adjuvant; 20 μg), HSP70 (antigen; 10 μg, Adjuvant; 20 μg), and GrpE (antigen; 10 μg, Adjuvant; 20 μg). Groups vaccinated with BCG (as positive control for the efficacy testing experiment of TB vaccine) were subcutaneously vaccinated with 3 × 10^5^ CFU of BCG Pasteur 1173 P2 at the time of the 2^nd^ immunisation using the antigens. Four weeks after the last immunisation, 5 mice per group were euthanized to analyse the immune responses, and adjuvant controls and vaccinated mice were aerogenically infected with Mtb K as previously described^52^. Briefly, mice were exposed to Mtb K for 60 min in the inhalation chamber of an airborne infection apparatus calibrated to deliver a predetermined dose (Glas-Col, Terre Haute, IN, USA). To confirm the initial bacterial burden, four mice were euthanised one day later, and approximately 150 viable bacteria were delivered to the lungs of each mouse. This *in vivo* protective efficacy test was conducted twice. At 4 and 8 weeks after the Mtb K challenge, single-cell suspensions from the spleen and lungs of BCG-, HSP70-, and GrpE-immunised mice were stimulated with antigens (PPD, HSP70, and GrpE; 10 μg/mL concentrations) for 12 h at 37 °C in the presence of GolgiStop (BD Biosciences, San Diego, CA, USA), and then, the populations of antigen-specific CD44^high^ T cells producing IFN-γ, TNF-α, and/or IL-2 were analysed by flow cytometry. Additionally, the cytokines released into in the culture supernatants 24 h after antigen stimulation at 37 °C in the absence of GolgiStop were quantified using ELISA.

### ELISA for serum IgG titres

Antigen-specific IgG1 (Sigma) and IgG2c (Southern Biotech, Birmingham, AL, USA) responses in serum from antigen-immunised mice were analysed by sandwich ELISA, as described previously^14^. Briefly, antigen-specific Abs were detected on 96-well plates coated with 1 μg/mL antigens, and blocked with PBST supplemented with 5% BSA. Serum samples diluted from 1/1000 were incubated for 24 h at 4 °C. To detect antigen-specific IgG1 and IgG2C, incubation with HRP-conjugated Abs was performed for 1 h at 37 °C. Optical densities (OD) were determined at 495 nm within 15 min of stopping the reaction.

### Colony forming units (CFU) and histopathology

At 4 and 8 weeks after the Mtb K challenge, the numbers of viable bacteria in the lung and spleen were obtained for CFU counts. Briefly, each bacterial count was determined by plating serial dilutions of the organ homogenates onto Middlebrook 7H10 agar (Becton Dickinson, Franklin Lakes, NJ, USA) supplemented with 10% OADC enrichment medium until the late-exponential phase. The numbers of colonies were counted after 3 weeks of incubation at 37 °C. The resultant values were reported as the mean log_10_CFU ± SD per gram of lung and spleen tissues. Lung samples collected for histopathology were preserved overnight in 10% normal buffered formalin, embedded with paraffin, cut into 4–5-mm sections, and stained with hematoxylin-eosin (H&E). A specialised pathologist evaluated the inflammation level in the tissue sections from the lung in a blinded manner.

### Intracellular cytokine staining and analysis of T cell subpopulations

Single cell suspensions from the spleen and lung were prepared as described preciously^14^. Single-cell suspensions (2 × 10^6^ cells) from adjuvant-, BCG- and antigen-immunised mice were stimulated with PPD (10 μg/mL), HSP70 (10 μg/mL), or GrpE (10 μg/mL) for 12 h at 37 °C in the presence of GolgiStop. Cells were first blocked with Fc Block (anti-CD16/32; eBioscience, San Diego, CA, USA) for 15 min at 4 °C and then stained with fluorochrome-conjugated Abs to Live/Dead (InvivoGen, San Diego, CA, USA), CD3 (BD Biosciences), CD4 (eBioscience), CD8 (eBioscience), CD62L (eBioscience), CD44 (eBioscience), and CD127 (eBioscience) for 30 min at 4 °C. Cells stained with the appropriate isotype-matched immunoglobulin were used as negative controls. After they were washed with PBS twice, cells were fixed and using a Cytofix/Cytoperm Kit (BD Biosciences) according to the manufacturer’s instructions. Intracellular TNF-α (eBioscience), IL-2 (eBioscience), and IFN-γ (eBioscience) were detected with fluorescein-conjugated Abs in a permeation buffer. Cells were analysed using a FACSverse flow cytometer and commercially available software (FlowJo).

### Cytokine measurements

The levels of cytokines, such as IL-17, IL-5, TNF-α, IL-10, IL-2, and IFN-γ, were measured by ELISA, according to the manufacturer’s instructions (eBioscience).

### Statistical analysis

All analyses were repeated at least two times with consistent results. The levels of significance for comparisons between samples were determined by Dunnett’s post-test or unpaired *t*-tests using statistical software (GraphPad Prism, version 5; San Diego, CA, USA). Results are expressed as means. Values of *p < 0.05, **p < 0.01, and ***p < 0.001 were considered statistically significant.

## Electronic supplementary material

Supplementary Information
