# Supplementary material for: Comparison of immunogenicity and vaccine efficacy between heat-shock proteins, HSP70 and GrpE, in the DnaK operon of Mycobacterium tuberculosis
Source: Sci Rep. 2018 Sep 26;8:14411. doi: 10.1038/s41598-018-32799-z (PMC6158166; doi:10.1038/s41598-018-32799-z)
Supplement: Supplementary file 1 — Supplementary Information [file 41598_2018_32799_MOESM1_ESM.pdf]

Comparison of immunogenicity and vaccine efficacy between heat-shock proteins, HSP70 and GrpE, in the DnaK operon of *Mycobacterium tuberculosis*

Woo Sik Kim[1,2†], Jong-Seok Kim[1†], Hong Min Kim[1], Kee Woong Kwon[1], Seok-Yong Eum[3], Sung Jae Shin[1*]

[1]Department of Microbiology, Institute for Immunology and Immunological Disease, Brain Korea 21 PLUS Project for Medical Science, Yonsei University College of Medicine, Seoul, South Korea, [2]Advanced Radiation Technology Institute, Korea Atomic Energy Research Institute, Jeongeup, South Korea, [3]Division of Immunopathology and Cellular Immunology, International Tuberculosis Research Center, Changwon, South Korea

[†] These authors contributed equally to the work.

*Correspondence:
Sung Jae Shin
E-mail address: sjshin@yuhs.ac

Supplementary Fig. S1. Flow cytometry analysis of multifunctional T cells.

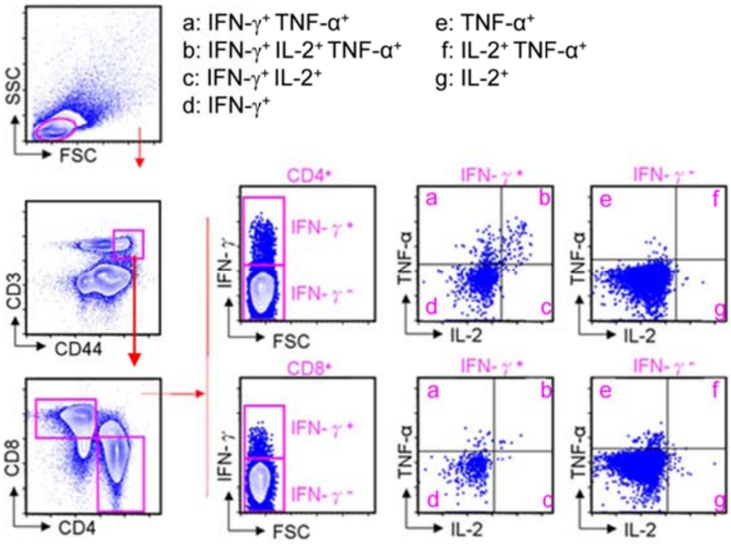

Spleen and lung cell suspensions were analysed by multiparameter flow cytometry for cytokine production, and data were collected on FACSverse flow cytometer with subsequent analysis using FlowJo software. For the analysis of cytokine producing T cells, an inclusion gate was drawn around cells with equivalent forward scatter-height and forward scatter-area values to exclude doublets and larger cell aggregates. Lymphocytes were then gated based on their characteristic pattern of forward scatter and side scatter, and dead cells were excluded by gating out cells that stained positive with the LIVE/DEAD viability dye (data not shown). Lymphoid gates were then drawn around $CD3^+$ cells to identify all T lymphocytes, followed by gating on $CD4^+CD44^{high}$ and $CD8^+CD44^{high}$ respectively to limit the analysis of cytokine producing cells to T lymphocytes. In order to distinguish multifunctional T cell subsets, gates were first delineated for positive staining for each cytokine using non-stimulated controls from matched animals to determine background staining. As shown in Fig. 4 and Fig. 5, these data were then used to delimit production of each of the three cytokines by every gated $CD44^{high}$ T cells, and the proportion of total events constituting each cytokine producing subset was determined. Values were converted to absolute numbers by multiplying these proportions by the total cell yield obtained from the lungs of each animal.
